# General practitioners’ perceptions and experiences of knee osteoarthritis management: a qualitative study of barriers and facilitators to delivering guideline-recommended treatments

**DOI:** 10.1080/02813432.2026.2688368

**Published:** 2026-07-04

**Authors:** Astrid Maria Carolina Trulsson, Torsten Risør, Susanne Reventlow, Søren Thorgaard Skou, Anne Møller

**Affiliations:** a Research Unit for General Practice and Section of General Practice, Department of Public Health, University of Copenhagen, Copenhagen, Denmark; b The Research and Implementation Unit PROgrez, Department of Physiotherapy and Occupational Therapy, Central and West Zealand Hospital, Slagelse, Denmark; c Center for Muscle and Joint Health, Department of Sports Science and Clinical Biomechanics, University of Southern Denmark, Odense, Denmark; d Department of Research, Central and West Zealand Hospital, Slagelse, Denmark

**Keywords:** Knee osteoarthritis, primary health care, general practitioners, guideline recommended care, non-surgical treatment, referral pathways, qualitative research

## Abstract

**Background:**

Knee osteoarthritis (OA) is a prevalent condition and public health concern. Guidelines recommend non-surgical treatments – such as education, exercise and weight management – as core elements of OA management; however, uptake remains low. General practitioners (GPs) play a crucial role in delivery, yet little is known about how GPs navigate OA treatments in the Scandinavian context.

**Aim:**

To explore Danish GPs’ perceptions and experiences of knee OA management, focusing on treatments and referral, and to identify key barriers and facilitators to delivering guideline-recommended treatments.

**Methods:**

Twelve semi-structured online interviews were conducted with GPs and GP trainees. Participants were recruited through a combination of convenience and self-selection sampling. Data were analyzed inductively using thematic analysis.

**Results:**

Four themes were identified: (1) knowledge and practice extend beyond guidelines, (2) non-surgical treatments are limited and inconsistently provided, (3) conceptual and relational tensions are negotiated and (4) fostering patient motivation is challenging.

**Conclusion:**

GPs displayed generally up-to-date knowledge of core non-surgical treatments, although familiarity with referral criteria was more variable. They held positive attitudes toward exercise and physiotherapy-led treatment, but experienced limited active dissemination of guidelines. Structural barriers, including fragmented pathways and uneven municipal services limited delivery of non-surgical treatments. Weight loss was viewed as effective in relieving joint pain but an unsustainable treatment focus, highlighting the need for a feasible, weight-inclusive approach to weight management. Strengthening cross-sector coordination, developing and implementing multidisciplinary OA services as well as clarifying where responsibility for fostering patient motivation should be placed, may enhance delivery of non-surgical care.

## Background

Osteoarthritis (OA) affects 595 million people worldwide and is a leading cause of pain and disability [1]. Knee OA is the most common type of OA and its burden has increased markedly over recent decades in the Nordic region [1,2]. In Denmark, ∼60,000 patients consult general practice with symptoms of knee OA annually [3]. As primary care providers, general practitioners (GPs) are instrumental in the management of knee OA. GPs are often the first point of contact, responsible for management and coordination of further care [4].

National^1^ and international knee OA management guidelines recommend non-surgical treatments – including education, self-management, therapeutic exercise and when appropriate, weight management – as core treatments [3,5–7]. Knee replacement surgery is typically considered when core and selected adjunctive treatments – such as pharmacological therapies, assistive aids or bracing – fail to relieve pain or improve function [3,8].

Studies report low rates of core OA treatments in primary care, alongside high rates of imaging, medication use and surgical referral [9–13]. A cross-sectional Danish study found that only about one-third of knee OA patients referred for orthopedic consultation felt informed about their condition, with a similar proportion having consulted a physiotherapist in the past year [14]. Likewise, another study reported that only 23% of patients had received the recommended combination of exercise, education and dietary guidance before orthopedic referral [15].

Barriers to guideline-recommended care exist at patient, provider and system levels. Reported GP-provider barriers commonly include uncertainty about the effectiveness of exercise therapy and perceived limited knowledge of exercise prescription [16–19]. Patient-related factors, such as preferences for surgical treatments and low motivation for exercise, complicate care delivery [20–22]. Finally, system-level challenges – such as limited access to affordable physiotherapist-led exercise and unclear referral pathways – further hinder delivery of recommended care [18,23].

While several studies have explored GPs’ knowledge and attitudes toward knee OA management across different healthcare systems, most originate from the North American, Australian and New Zealand contexts [17,18,24–27], with a limited number of studies from European settings [28]. Existing research differs in focus and scope, with some exploring GP perspectives specifically whilst others include a broad range of healthcare professionals or patient viewpoints.

## Aims

We aimed to (i) explore GPs’ perceptions and experiences of knee OA management, focusing on treatments and referral in general practice and (ii) identify factors shaping the delivery of guideline-recommended treatments, including barriers and facilitators.

## Material and methods

### Study design and setting

This qualitative interview study follows the COREQ reporting criteria [29]. Seven GPs and five GP trainees in Denmark were interviewed. General practice forms the cornerstone of Danish primary care within a tax-funded healthcare system organized at national, regional and municipal levels [30]. Most residents are listed with a GP, who acts as the primary point of contact and gatekeeper to specialist and hospital services [31]. Elective surgery is free of charge in the public sector, but patients can access private services through insurance, public funding schemes and out-of-pocket payments. Recent hospital sector reforms have centralized services and shifted the provision of care across hospitals, likely influencing referral pathways between GPs and specialists [30].

### Participants and recruitment

We sought variation in age, sex and practice location (urban/rural). Participants were selected using a mixed recruitment strategy:

- self-selection: postal invitation to 200 practices in two Danish regions, selected alphabetically from a public register;
- convenience sampling: Facebook posts in two closed groups for GPs (268 members) and physicians (9000 members); and
- self-selection: online notice on the digital news page of the University of Copenhagen’s Section of General Practice.

Most postal invitees did not respond. All who expressed interest were interviewed and none withdrew. The final sample comprised seven GPs and five GP trainees (seven from the Capital Region, five from Region Zealand), aged 31–69 years. We define a GP trainee as a physician who has completed medical school and is enrolled in the 4.5-year specialist training program in general practice. Participants received financial compensation (138.87 DKK ≈ €19 per 10-minute module) in line with regional guidelines (Table 1).

**Table 1.** Demographic characteristics of participating GPs and GP trainees.

Pseudonym	Position	Gender	Age	Years since medical graduation
Amelie	GP trainee	F	30–39	<5
Daniel	GP	M	60–69	40–50
Elena	GP	F	40–49	20–30
Emma	GP trainee	F	30–39	5–10
Eva	GP	F	40–49	10–20
Henrik	GP	M	50–59	10–20
Isabella	GP trainee	F	30–39	5–10
Laura	GP trainee	F	30–39	<5
Mira	GP	F	60–69	30–40
Olivia	GP trainee	F	30–39	5–10
Sofia	GP	F	40–49	10–20
Thomas	GP	M	50–59	20–30

### Data collection and analysis

Interviews were conducted from January–July 2022. As part of patient and public involvement, a patient representative with knee OA (recruited *via* the Danish Rheumatism Association) took part in a one-hour preliminary interview and provided input on relevant topics for the interview guide. The guide covered various aspects of knee OA management in general practice and was piloted with one GP. To encourage open reflection and minimize bias, participants were invited to share their thoughts and approaches to knee OA management, without explicitly framing the interview around adherence to guidelines. Specific and open-ended questions facilitated free expression of experiences, and the guide was iteratively revised to reflect preliminary findings and support ongoing development of the analysis [32]. Prompts were used *ad hoc* to clarify and expand responses.

The research team comprised a clinical MD and PhD fellow (AMCT); two clinical GPs and associate professors in general medicine (AM and TR), one of whom is a practice owner (AM); a clinical GP, anthropologist and professor in general practice (SR); and a physiotherapist and professor of exercise and human health with over 10 years of research experience in knee OA (STS). Interviews were conducted by the first author (female MD/PhD fellow with qualitative research training from the Liverpool School of Tropical Medicine and the University of Copenhagen). Participants were informed about the interviewers’ position and background, as well as the aim of the project. The multidisciplinary team regularly discussed emerging findings to challenge assumptions, integrate perspectives and refine the thematic framework. No prior relationship existed between interviewer and participants.

Due to ongoing COVID-19 precautions, all interviews were conducted online one-to-one in Danish *via* Microsoft Teams, typically with participants joining from their home or clinic. Each lasted about an hour and was video-recorded. No repeat interviews were conducted. The interviewer took brief field notes during and after sessions to capture contextual impressions and emerging ideas. Sample size was guided by the concept of information power, which takes into account the width of the study aim, specificity of the participants’ experiences and knowledge, use of theory, strength of interview dialogue and type of data analysis [33]. While the exploratory nature of the study and use of cross-case analysis supported inclusion of a broader sample, the specificity of the participant group and richness of the interview data contributed to sufficient information power. We therefore considered 12 interviews adequate for the study aim. A research assistant transcribed the interviews verbatim; transcripts and findings were not returned to participants. Quotations presented in this article were translated into English by the first author.

Data were analyzed inductively to explore patterns and meaning across the dataset, using Braun and Clarke’s reflexive thematic analysis [34]. All the transcripts were read by AMCT, and selected transcripts were read by TR and SR, to support active interpretation of patterns and meanings across the dataset. AMCT coded all transcripts manually and with NVivo software. Codes were generated inductively and iteratively grouped into categories, which were developed and refined into themes through repeated engagement with the data and team discussions. Transcripts were re-read as part of this interpretive process. As GPs and GP trainees expressed similar views, their data were analyzed jointly and are referred to collectively as ‘GPs’ or participants throughout this article.

### Ethical considerations

The study was registered in Region Zealand’s Research Registry (REG-127-2021). According to Section 14(2) of the Danish Act on Research Ethics Review (Komitéloven), interview studies that do not involve collection of biological material are exempt from ethics committee approval. This was confirmed by the Danish National Committee on Health Research Ethics (Den Nationale Videnskabsetiske Komité, NVK) and the Danish Regional Committees on Health Research Ethics (De Videnskabsetiske Medicinske Komitéer, VMK) (case no. MN-2025-01604). Participants provided written and verbal informed consent before interviews. All data were anonymized and handled in accordance with data-protection regulations.

## Results

The analysis generated four themes important for shaping the delivery of guideline-recommended treatments in general practice: (1) knowledge and practice extend beyond guidelines, (2) non-surgical treatments are limited and inconsistently provided, (3) conceptual and relational tensions are negotiated and (4) fostering patient motivation is challenging.

### Knowledge and practice extend beyond guidelines

While GPs demonstrated knowledge of core non-surgical treatments, familiarity with specific guideline recommendations and referral criteria appeared more variable. Their practice often extends beyond national guidelines.

All participants described non-surgical treatments as central to knee OA management, typically including patient information, exercise and physiotherapist-led exercise as well as weight management and pain medication when needed. However, their confidence in and emphasis on specific components – particularly weight management – varied. Whilst all GPs encourage patients to exercise, the majority do not feel comfortable instructing specific exercises for knee OA. Most believe physiotherapist-led exercise offers more in-depth information about the condition and provides greater benefits than GP guided exercises or patient-led exercise alone. Most GPs value end-of-treatment reports from physiotherapists and use them in further management.

[Physiotherapists] have the time and the tools—and maybe the patients have dressed in more suitable clothes that day.[…] But for me, it’s a 15-minute slot where I need to examine, explain what’s happening in the knee and why they have symptoms, and write notes - there just isn’t enough time [to instruct in specific exercises]. I’m also not sufficiently trained to. – Sofia

Beyond recommended non-surgical treatments, some GPs also draw on alternative treatments whilst simultaneously informing patients about the lack of scientific evidence for such therapies. This pragmatic approach is used to maintain patient trust, support patient motivation and navigate limited availability of non-surgical treatments. For instance, some GPs recommend acupuncture due to positive patient feedback and its availability at private physiotherapy clinics.

This pragmatic orientation also shapes how GPs relate to national guidelines, which are often seen as distant from everyday practice. Not considering themselves the key audience of national guidelines, the majority of GPs report having not read them. They are seen as lengthy, considering the many conditions GPs manage on an everyday basis. Instead, many rely on collegial networks and peer discussions to stay informed. Although GPs are familiar with core treatment principles, many remain uncertain about threshold-type rules for referral acceptance. Multiple GPs do not perceive that updated referral criteria is being effectively disseminated to general practice – particularly the requirements for completed physiotherapist-led exercise and recent radiographic imaging prior to orthopedic referral of knee OA that were introduced a couple of years ago [3]. Accordingly, changes to referral practice result from rejected referrals and peer discussions with GP colleagues. Several GPs call for explicit feedback on rejected referrals, as opposed to generic statements referring to national guidelines.

So what happens is that the hospitals change the way they assess referrals […]. In the middle of all this, you’re trying to help your patient move forward, but suddenly, the conditions for doing so change drastically. […] I think it would make sense if there was a collaboration around this. – Thomas

GPs emphasize the need for more structured communication channels, such as interdisciplinary forums, to actively communicate care changes and regional GP consultants to involve GPs in decision-making.

### Non-surgical treatments are limited and inconsistently provided

GPs describe limited and inconsistent availability of core non-surgical treatments, shaped by variation across municipalities, strict eligibility criteria and fragmented care pathways. They report uncertainty about which municipal services are available for weight management and physiotherapist-led exercise, particularly when serving patients from multiple municipalities. Patients often remain ineligible for dietician referral unless pre-diabetic or exceeding a specific body mass index. Some also note the lack of a user-friendly overview of local treatment options.

I always feel like I have to search for the [dietician referral] criteria—I think they’re constantly changing. Sometimes there’s an offer available for [knee OA patients] and other times there isn’t, so I often feel like I’m hitting a wall in that regard. And then, of course, the patient must pay for it themselves and… it’s expensive. – Emma

Most GPs perceive multiple barriers to physiotherapist-led exercise, such as transport difficulties, a lack of services outside normal office hours for patients in the workforce, long waiting times and, for some patients, self-payment for municipal treatments. Some GPs reflect on the paradox of orthopedic surgeons accessing referral to free municipal physiotherapist-led exercise, whereas this is not consistently available to GPs.

I work in a relatively low-income area, so I encounter economic barriers. I would be glad if there were some municipal treatment offers. It would be great if I could tell patients, ‘call the municipality, they start a group every two weeks or once a month, and you can join.’ But as it is now… it’s expensive, isn’t it? – Henrik

GPs observe that patients with fewer economic or personal resources, are easily lost in current pathways, which require navigating multiple providers, such as dieticians, physiotherapists and radiography services. Some suggest developing cohesive multidisciplinary services provided at municipal level that combine patient education, weight management and physiotherapist-led exercise.

It’s my impression that patients have very different experiences and very different paths into the system. [Knee OA is a major] health condition, […] I think it would be fairly easy to set up a program that provides structured [patient education, exercise and weight management]. – Isabella

### Conceptual and relational tensions are negotiated

GPs describe navigating tensions in knee OA management, particularly around weight management, surgical eligibility and referral processes. These areas often create uncertainty in how best to balance recommended treatments with patient circumstances. Many GPs believe it is important to reduce the load on the joint and that losing weight will help relieve pain and routinely inform patients about its benefits. However, most consider it an unsustainable treatment, describing how patients often struggle to lose weight, tend to regain it over time and require more continuous support and supervision than general practice can provide. Several GPs question the practicality of weight loss as a standalone intervention, noting that while it makes sense to decrease the load on the joint, it rarely aligns with patients’ everyday realities. Consequently, many view weight-inclusive approaches – focusing on healthy behaviors rather than weight loss – as more sustainable alternatives to weight-centered treatment.

I think the focus on weight-inclusive health is starting to make so much sense! I haven’t really found a solid position when it comes to patients who weigh far too much for their knees. Because it doesn’t help to tell them to lose weight—[…] and I don’t believe they can. – Eva

Similarly, while GPs view surgery as a last resort for knee OA patients (when symptoms are disabling and non-surgical treatments insufficient), referral is often shaped by both patient preferences and the need to preserve the doctor–patient relationship.

Ideally, I prefer to refer them when I genuinely believe there is [an orthopedic indication] for it. But sometimes, you have to admit defeat and say, I’m not getting any further with this, and if we are to maintain a good relationship going forward, I also have to say that I’m not able to help you, so let’s see if someone else can. – Laura

Several GPs also described uncertainty around surgical evaluation due to inconsistencies in referral processes and thresholds for total knee arthroplasty over time, across hospitals and between public and private sectors. They noted that centralized referral systems^2^ have made criteria less transparent, resulting in a loss of locally grounded knowledge previously developed through experience with consistent referral pathways and familiarity with individual orthopedic departments’ preferences. This contributes to a feeling of ambiguity about when patients are candidates for surgery.

You can ask how much osteoarthritis needs to be present for a surgeon to decide on surgery? […] This threshold has shifted over time […] There may be economic considerations behind it. […] There has been an ongoing debate about the appropriate level of arthroscopies, MRI scans and surgeries, and it’s clear that practices [vary]. – Thomas

### Fostering patient motivation is challenging

Those who come with COPD, you have to tell them to stop smoking […] It’s something you have to take away from them. [In knee OA management], it’s something you have to get them to do. [i.e. exercise] I don’t know what is the hardest. It could be equally hard. – Laura

GPs describe patient motivation and engagement as central in regards to knee OA management, but also one of its greatest challenges. One GP contrasted the challenges of promoting behavioral change across conditions, describing how smoking cessation in COPD involves asking patients to give something up, whereas knee OA management requires encouraging patients to take on new behaviors. The contrast between treatments which require sustained patient participation and behavioral change over time, such as exercise, weight management and physiotherapist-led programs and treatments, which involve a different and more time-limited form of patient engagement, such as medication or surgery is also highlighted.

It is a big challenge that part of the treatment actually consists of something the patient has to do themselves and there are no results that come from one day, week, or month to the next. It’s something that requires quite a lot of effort. It’s a challenge. It requires a patient who is really motivated. It requires patient resources, that’s the challenge for the doctor. I can give all the good advice I can think of, but if they don’t follow it, nothing happens and then both them and I are frustrated. – Laura

Many GPs also reflect on who should take responsibility for fostering motivation and providing in-depth information about knee OA. While they agree that supporting patient motivation is essential, they often feel constrained by short consultations and limited expertise in exercise instruction. As a result, they view physiotherapists – and structured municipal programs – as better placed to deliver sustained encouragement and guidance than GPs alone.

It seems, within the hospital system [it is believed] it’s a GP task to get people to lose weight. And I often sit with the feeling that, yes, I can tell patients how to change their diet so they can lose weight, but they just come back again. So, something is missing—I can’t figure out whether it’s acceptance that this isn’t a treatment that works or whether there’s a missing system to keep them going if that’s the strategy we’re supposed to use. – Isabella

Pain sometimes acts as a barrier to exercise engagement. As pharmacological options are limited and carry risks, some GPs also refer to private orthopedic practice to reinforce advice and administer intra-articular steroid injections to help patients break a negative pain cycle and enable participation in physiotherapy.

## Discussion

Across the four themes, three interrelated dimensions were particularly salient: (1) knowledge of core treatments generally aligns with guideline recommendations, although guideline dissemination appears limited and task distribution remains unclear, (2) structural constraints and conceptual tensions limit practical implementation and (3) patient motivation is challenging and responsibility for sustaining engagement remains unclear. These findings highlight challenges at the interface between clinical care and system organization, with implications for cross-sector collaboration, service planning and the development of future guidelines and task distribution in primary care.

Knowledge of core treatments generally aligns with guideline recommendations, although guideline dissemination appears limited and task distribution remains unclear.

Our findings suggest that GPs demonstrate knowledge of core treatment principles aligned with guidelines [18,21,24,38], although familiarity with the guideline documents themselves is more limited. GPs’ ambivalence toward the national guideline appears to reflect both its format and intended audience, with lengthy documents perceived as poorly tailored to general practice and everyday patient care – a finding echoed elsewhere [39]. In line with recent studies, GPs perceived exercise and physiotherapy-led treatment as effective [18,21,24,25,38]. However, awareness of referral criteria was uneven and GPs primarily reported gaining knowledge of guideline-recommended treatments through collegial exchange rather than systematic top-down dissemination. Participants emphasized the need for clearer communication of referral criteria and for stronger GP involvement in how such information is developed and conveyed to general practice.

Many GPs reported limited time, confidence and scope to instruct patients in knee-specific exercises [18,19,26], preferring instead to refer to physiotherapists [17]. Physiotherapists’ treatment summaries were valued and supported continuity, a finding echoed in previous studies [25]. Given that exercise therapy is a core physiotherapy competency, granting patients direct, free access to this treatment may increase uptake. At the same time, enhancing GPs’ capacity to prescribe or demonstrate basic exercises could still benefit patients unable to access physiotherapist-led programs.

### Structural constraints and conceptual tensions limit practical implementation

Our findings suggest that for guidelines to influence practice, they must not only be accessible but fit into existing healthcare systems and align with the experiential knowledge shaping how GPs and patients engage with care.

Uneven provision of physiotherapy and weight management services across municipalities made treatment pathways difficult to navigate. Similar findings have been reported in countries with publicly financed healthcare, where non-surgical OA services remain non-standardized and unevenly distributed [40,41]. Such variation risks reinforcing inequalities, as patients with greater resources or higher health literacy may be better equipped to navigate the system. In addition, practical issues such as cost, transport, waiting times and limited service hours shaped management [18,21,25,27]. Also, as echoed in other studies, limited awareness of referral pathways hinders the delivery of non-surgical care [18,21,23,24,27].

Several GPs questioned the feasibility and sustainability of weight loss, consistent with evidence of frequent weight regain over time [42]. This highlights the importance of aligning recommendations with realistic, patient-centered goals. Previous studies describe GPs adopting a more holistic stance toward weight management [18,27,38] while low referral rates to dieticians may reflect financial barriers or skepticism about the long-term effectiveness of weight reduction [21,24]. Recent Danish data likewise show that dietary advice is seldom applied in knee OA management [15], echoing international findings; for instance, only around 1% of knee OA patients in the Netherlands received weight-loss advice from their GP [13]. Some participants reflected that weight loss might be more feasible if patients received tailored guidance, continuity of care and support to integrate changes into daily life.

Our findings reveal an emerging weight-inclusive health perspective among GPs, representing a novel and potentially sustainable alternative and marking a shift in how weight is conceptualized in knee OA management in general practice. While GPs acknowledged the potential benefits of weight reduction for relieving joint symptoms [43], many emphasized focusing on healthy lifestyle change rather than weight loss itself [27]. They perceived weight loss as difficult to initiate and sustain in general practice. This illustrates a conceptual tension in weight management: balancing the potential benefits of weight loss with the recognition that sustainable health may be better supported through lifestyle-focused, weight-inclusive care.

### Patient motivation is challenging and responsibility for sustaining engagement remains unclear

A perceived lack of patient motivation for core treatments suggests a need for greater focus on how patients can best be supported in sustaining behavioral change. GPs often describe frustration and a sense of defeat when patients are unmotivated to pursue recommended treatments, pointing to a broader feeling of powerlessness. Similar difficulties in sustaining patient motivation for lifestyle-based treatments, particularly exercise and weight management have been highlighted in other studies [17,18,21,23,25,27,44].

Health professionals play a decisive role in motivating patients and studies illustrate how patients place different expectations on different caregivers [25,28,41,45]: with some patients trusting orthopedic surgeons for initial guidance, physiotherapists for diagnosis and exercise and GPs as coordinators of their general health [25]. Similar patient expectations were described by GPs in our study and appeared to shape adaptive physician behaviors, such as occasional strategic usage of referrals to reinforce non-surgical care or to sustain doctor–patient trust. This invites discussion on where responsibility for motivational tasks related to behavioral change are best placed within the healthcare system.

## Implications and future directions

Structural, conceptual and motivational factors shape the delivery of core treatments in general practice. Improving dissemination and involving GPs more actively in guideline development and implementation may strengthen cross-sector collaboration and reduce administrative friction in knee OA care. Likewise, clearer communication of referral criteria may improve guideline uptake [46] and reduce referral rejections. Referral pathways to secondary care can be optimized by improved two-way communication between sectors [46], yet further research is warranted to explore the most effective strategies across healthcare settings [47]. Additionally, user-friendly digital overviews of municipal treatment options may help support GP navigation.

Furthermore, GPs proposed developing multidisciplinary services at the municipal level that integrate patient education, physiotherapist-led exercise and weight management to provide cohesive support [28,41]. Initiatives, such as the Osteoarthritis Chronic Care Program in Australia, a multidisciplinary non-surgical care model promoting whole person assessment, demonstrate that coordination of care is both feasible and effective, underscoring the relevance of these suggestions [48].

Moreover, clarifying where motivational responsibilities lie – whether in general practice, physiotherapy or multidisciplinary municipal services – could help sustain engagement while easing the burden on individual GP consultations. Hence, a clearer allocation of tasks across the healthcare system – aligned with expertise and scope of practice – may strengthen uptake of physiotherapy-led exercise and weight management.

At the clinical level, addressing the tension related to weight management – balancing the benefits of weight loss with the feasibility of weight-inclusive approaches – may strengthen practitioner and patient engagement. Engagement is more likely to improve when care aligns with what matters to patients and is workable in everyday life [49].

Our findings indicate that not all departures from guideline recommendations reflect deficiencies in care. In some cases GPs adapted advice to what was considered more feasible or sustainable, referred strategically to reinforce non-surgical care or acknowledged complementary, non-evidence-based treatments valued by patients [18,21,24]. Such pragmatic adaptations illustrate how patient-centered care may require flexibility beyond the guideline and underscore the importance of distinguishing adaptive tailoring from barriers that may genuinely limit the uptake of core treatments.

Although this study focused on treatment delivery, other factors not explored in depth in this analysis may also contribute to the care gap. Important aspects, such as GPs’ perspectives on the diagnostic process, use of imaging and communication of diagnostic uncertainty were less prominent in this analysis and warrant further investigation. Moreover, both GPs’ and patients’ beliefs about knee OA, as well as their expectations of care, are likely to influence management decisions. Future research should address these dimensions to provide a more comprehensive understanding of the challenges and opportunities in improving knee OA management in general practice.

## Strengths and limitations

This study’s semi-structured interview format allowed in-depth exploration of GPs’ perceptions of knee OA management, focusing on treatments and referrals. Our participant sample reflects age, sex and geographical demographics of Danish GPs. It is possible that selection bias favored participants with an interest in knee OA, displaying greater knowledge of management than the average GP. Furthermore, their knowledge of core non-surgical treatments may not necessarily be translated into practice behavior. Online interviews, including occasional technical disruptions, may have influenced interaction and limited nonverbal cues. On the other hand, the format likely facilitated participation by reducing logistical barriers.

The multidisciplinary composition of the research team was a strength, informing the design, data interpretation and reflexive dialogue throughout the study. Complementary perspectives offered insider understanding of primary care while also enabling critical distance, which helped identify and contextualize relevant issues. At the same time, GPs were strongly represented within the team, which, while enhancing clinical relevance, may have limited the diversity of disciplinary viewpoints. Reflexivity was supported through discussions and a conscious effort to remain open to unexpected insights throughout the study.

## Conclusions

This study identified key factors shaping knee OA management in general practice, including GPs’ knowledge of core treatments, dissemination of guidelines, structural and organizational constraints and challenges related to weight management and patient motivation.

GPs demonstrated knowledge of core non-surgical treatments, although familiarity with specific guideline recommendations and referral criteria varied. Dissemination of guidelines and referral criteria occurs mainly through collegial exchange rather than active dissemination. GPs emphasized the need for clearer cross-sector coordination and greater involvement in developing referral criteria. Structural barriers, such as fragmented referral pathways, uneven service provision across municipalities and practical obstacles including cost and transport – were perceived to impede the delivery of non-surgical treatments.

Finally, most GPs perceived weight loss as an unsustainable treatment focus, despite recognizing its benefits on joint symptoms, highlighting the value of a more feasible and health-oriented approach to weight management in general practice. Clarifying responsibility for fostering and sustaining patient motivation – whether within general practice, physiotherapy or coordinated municipal programs – may help sustain patient engagement. Ultimately, recognizing and working with, rather than against, the clinical realities of general practice – including limited dissemination channels, structural constraints and the gap between guideline ideals and practical feasibility – is key to strengthening knee OA care in primary care.

## Notes

1 The Danish national guidelines for knee OA (2012) are currently under revision yet remain broadly aligned with current international consensus on core non-surgical treatments, but predates more recent updates that emphasize behavioral-change strategies and implementation support [3].

2 In 2011, the Capital Region launched central visitation units for both somatic and psychiatric care as part of a broader administrative effort to standardize elective referral pathways [35–37]. This move reflected a general trend across regions toward increased coordination and central oversight of the Danish health care system [30].
